# Development of a Metagenomics-Guided Personalized Synbiotic Protocol for Children with Autism Spectrum Disorder: An Exploratory Case Series

**DOI:** 10.3390/nu18111694

**Published:** 2026-05-26

**Authors:** Shaohan Zhang, Kevin Liu, Leo Shi, Chuyao Yan, Alma Wang, Ashley Liu, Haiyi Guo, Alex Xie, Xue-Jun Kong

**Affiliations:** 1 Athinoula A. Martinos Center for Biomedical Imaging, Massachusetts General Hospital, Charlestown, MA 02129, USA; szhang61@mgh.harvard.edu (S.Z.); leoxshi@gmail.com (L.S.); ycy31907@gmail.com (C.Y.); almawang749@gmail.com (A.W.); newyorkgas777@gmail.com (A.L.); haiyi.guo@childrens.harvard.edu (H.G.); alexander.xie.0705@gmail.com (A.X.); 2 Fetal-Neonatal Neuroimaging and Developmental Science Center, Department of Newborn Medicine, Boston Children’s Hospital, 300 Longwood Ave., Boston, MA 02115, USA; kevinliu.bmb@gmail.com; 3 Department of Medicine and Psychiatry, Beth Israel Deaconess Medical Center, Harvard Medical School, Boston, MA 02215, USA

**Keywords:** autism spectrum disorder, gut–brain axis, microbiota, synbiotics, metagenomics, personalized microbiome intervention, precision psychiatry

## Abstract

Background/Objectives: Gut microbiota dysregulation has been increasingly implicated in the pathophysiology of autism spectrum disorder (ASD), yet clinical responses to standardized probiotic interventions remain inconsistent, likely reflecting substantial inter-individual variability in baseline microbiome composition, host–microbe interactions, immune tone, and metabolic function. Here, we present a pilot implementation of a metagenomics-guided, personalized synbiotic intervention in children with ASD using the Systematic Microbiome Assessment and Reconstruction Therapy (SMART) framework. Methods: Seven children (aged 5–12 years) underwent longitudinal fecal shotgun metagenomic profiling, and dietary habits, food sensitivities, and regional dietary background were recorded as contextual factors potentially influencing microbiome composition and response to intervention. Individualized synbiotic formulations were constructed based on microbial taxonomic composition and inferred functional capacity and iteratively refined over time. Gastrointestinal outcomes were assessed through caregiver-reported clinical observations, whereas behavioral changes were evaluated using standardized instruments. Results: Several participants demonstrated improvements in gastrointestinal symptoms and selected behavioral domains. Notably, in a subset of participants, improvements in gastrointestinal function preceded measurable behavioral changes. Conclusions: Although limited by a small sample size and lack of a control group, these findings provide preliminary evidence supporting the feasibility of implementing a metagenomics-guided personalized synbiotic framework in ASD and generate hypotheses for future investigation. This work presents a preliminary conceptual framework for integrating microbial composition and inferred functional profiling into individualized intervention design and highlights the potential value of microbiome-informed stratification in future studies of treatment response. Larger controlled studies with objective outcome measures are warranted to further evaluate feasibility, reproducibility, and potential clinical utility.

## 1. Introduction

Autism spectrum disorder (ASD) is a heterogeneous neurodevelopmental condition characterized by social communication deficits and restricted, repetitive behaviors [1]. Its prevalence has risen markedly over recent decades, now affecting approximately 1 in 31 children in the United States [2]. However, clinical manifestations vary substantially across individuals, with differences in symptom severity and comorbidities [3,4,5]. Clinically, individuals with ASD may exhibit impaired socioemotional interaction skills, such as atypical eye contact and impaired peer relationships; communication deficits, such as the stereotypical and repetitive use of language; restricted and repetitive behaviors and interests; and altered sensory responses, such as heightened or atypical sensitivity to tactile, olfactory, auditory, and other sensory stimuli [6,7,8]. This heterogeneity is evident not only at the behavioral level but also at the biological level; for example, the complex genetic architecture, comprising both common and rare variants, may influence neurodevelopmental pathways across individuals in distinct ways; atypical neurodevelopment and brain connectivity are associated with the phenotypic abnormalities characteristic of ASD; furthermore, immune and inflammatory dysregulation play a role, as do alterations in the gut–brain axis involving changes in microbial composition and metabolic profiles [9,10,11,12]. The combined effects of these genetic, neural, immune, metabolic, and gut microbiota mechanisms, along with other multiple overlapping pathways, potentially contribute to ASD-related phenotypes [13,14,15]. Despite increasing recognition of these biological mechanisms, effective biologically targeted treatments for core or associated ASD symptoms remain limited.

Gastrointestinal (GI) comorbidities are among the most common and clinically significant features of ASD heterogeneity, affecting up to 9–70% of children with ASD [16]. Typical gastrointestinal manifestations in children with ASD include constipation, diarrhea, abdominal pain, gas/bloating, painful bowel movements, and feeding-related issues, such as food selectivity or food refusal [17,18]. These symptoms may lead to physical discomfort, sleep disturbances, irritability, and aggression, or other behavioral dysregulation, potentially exacerbating caregiver-reported behavioral difficulties [19,20]. Collectively, these observations suggest a bidirectional biological interplay between peripheral physiological processes and central nervous system function.

Cross-sectional studies have identified differences in microbial diversity, taxonomic abundance, and functional metabolic pathways in individuals with ASD compared with neurotypical controls [11]. Although specific taxa vary across cohorts, dysbiosis patterns have been repeatedly observed [21]. Mechanistic models propose that gut microbiota may influence neurodevelopment and behavior through multiple interconnected pathways, including immune activation, altered production of microbial metabolites (e.g., short-chain fatty acids and tryptophan-derived metabolites), disruption of intestinal barrier integrity, and modulation of gut–brain signaling via vagal, endocrine, and inflammatory pathways [22,23,24,25,26]. These findings support the microbiota–gut–brain axis as a biologically plausible contributor to ASD-associated symptoms, though causal relationships remain incompletely established.

Microbiome-targeted interventions, such as probiotics, prebiotics, and synbiotics, have therefore been investigated as potential therapeutic strategies [27]. However, clinical trials using standardized probiotic formulations have produced variable results. Some studies report improvements in GI symptoms and selected behavioral measures, whereas others demonstrate limited or inconsistent benefit [28]. For instance, Arnold et al. conducted a clinical trial utilizing the probiotic VISBIOME to treat children with ASD accompanied by gastrointestinal symptoms; they found that the probiotic treatment significantly alleviated specific symptoms of gastrointestinal discomfort selected by parents, although the primary outcome measures regarding gastrointestinal quality of life did not reach statistical significance [29]. Similarly, Santocchi et al. conducted a trial of a multi-species probiotic formulation in preschool-aged children with ASD; although there were no significant differences in symptom severity between the probiotic and placebo groups, certain gastrointestinal symptoms showed greater improvement [30]. These results likely reflect substantial inter-individual heterogeneity in baseline microbial composition and host–microbe interactions. Because microbial ecosystem responses are shaped by existing community structure and metabolic context, uniform formulations may not adequately address patient-specific dysbiosis profiles.

Advances in whole-genome shotgun metagenomic sequencing now enable high-resolution characterization of microbial taxonomic composition and functional gene capacity [31]. These advances enable a shift from empiric microbiome modulation toward precision, microbiome-informed intervention strategies. By identifying individualized dysbiosis patterns and functional deficits, targeted synbiotic formulations may be designed to restore ecological balance and potentially influence downstream immune and metabolic pathways.

To address this gap, we developed a metagenomics-guided personalized synbiotic protocol designed to account for inter-individual variability in microbial composition. Within an exploratory case-series framework, children with ASD underwent baseline fecal metagenomic sequencing to inform individualized synbiotic selection and longitudinal adjustment. We aimed to evaluate the feasibility and tolerability of this personalized microbiome-guided intervention and to explore preliminary changes in gastrointestinal and behavioral outcomes. Although not designed to establish efficacy, this exploratory study provides a mechanistically informed and precision-oriented framework for microbiome-based intervention in ASD and informs the design of future controlled trials.

## 2. Methods

### 2.1. Ethics Approval

The study protocol was reviewed and approved by the Medical Research Ethics Committee of Hebei Children’s Hospital (Approval No. 219). The committee determined that the study design adequately protected participants’ health, rights, and privacy, and that potential risks and harms were minimized. The original ethical approval was granted on 29 January 2021, and the continuation of the project was approved on 28 June 2023.

### 2.2. Metagenomic Sequencing and Bioinformatics Analysis

In this study, MetaPhlAn4 (Version 4.0.0) was used to profile microbial taxa and analyze relative abundances in metagenomic sequencing data [32]. Raw sequencing reads were first subjected to quality control to remove adapter sequences, low-quality reads, and host-derived contaminations, resulting in high-quality filtered reads. The filtered reads were then aligned to species-specific marker genes in the built-in MetaPhlAn4 database using the integrated Bowtie2 (Version 2-2.5.4) alignment algorithm. Based on the alignment results, relative abundances of microbial taxa were estimated through MetaPhlAn’s marker gene-based statistical framework, enabling accurate taxonomic annotation from the kingdom to the species level. This approach improves detection sensitivity for low-abundance species and reduces false-positive assignments, providing a robust basis for characterizing gut microbiome composition.

Metagenomic assemblies were subsequently used to predict open reading frames (ORFs), and the corresponding amino acid sequences were extracted for functional annotation. Functional annotation was performed using the KEGG (Kyoto Encyclopedia of Genes and Genomes) database to characterize metabolic potential and pathway distribution. In addition, published studies on microbial metabolic interactions in the human gut were referenced to integrate microbial taxonomic profiles with metabolic pathways, enabling integrated mapping from community composition to functional capacity [33,34]. Relative abundances of metabolic pathways and functional modules were calculated to construct the metabolic landscape of each sample. Shotgun metagenomic sequencing enabled simultaneous characterization of microbial taxonomic composition and functional metabolic potential. The resulting functional indicators were then compared with a reference population database to determine their percentile positions within a healthy pediatric cohort. The reference dataset comprised over 400 gut metagenomes from healthy children aged 3–18 years and was used to establish percentile-based functional baselines for pediatric microbiome analysis.

### 2.3. Systematic Microbiome Assessment and Reconstruction Therapy (SMART)

**Figure 1:**
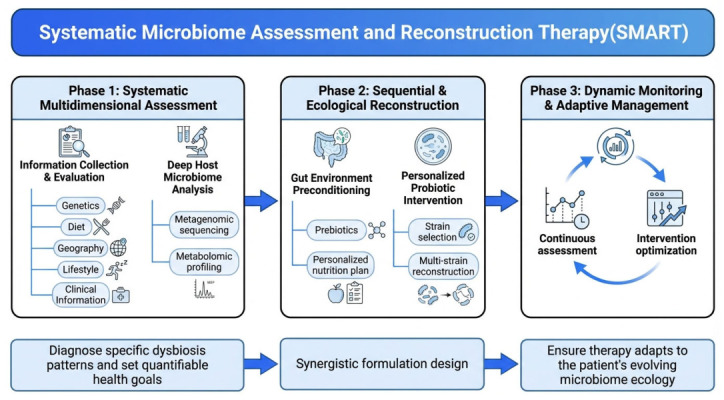
Conceptual overview of the Systematic Microbiome Assessment and Reconstruction Therapy (SMART) framework.

The intervention strategy was guided by the Systematic Microbiome Assessment and Reconstruction Therapy (SMART) framework. This framework elevates personalized probiotic intervention from an isolated product supplement to a comprehensive treatment paradigm integrating multi-omics assessment, in-depth host context analysis, ecological rational reconstruction, and dynamic monitoring. As illustrated in Figure 1, the SMART framework consists of three sequential phases, including assessment, ecological reconstruction, and adaptive monitoring. Artificial intelligence (AI) was used to support multidimensional data integration, predict strain-host compatibility, and optimize intervention protocols.

The SMART framework consists of three sequential phases:

1. Systematic Multidimensional Assessment, integrating host metadata and metagenomic profiling to characterize dysbiosis patterns.
2. Sequential Ecological Reconstruction, involving gut environment preconditioning and personalized probiotic intervention through function-matched strain combinations.
3. Dynamic Monitoring and Adaptive Management, in which continuous microbiome assessment guides optimization of intervention strategies.

This framework enables precision microbiome interventions by integrating host context, microbial community structure, and functional metabolic capacity.

### 2.4. AI-Assisted Knowledge-Base Construction and Regimen Drafting

To support clinician interpretation during individualized synbiotic design, a knowledge base was constructed from two sources: (i) a systematic review of published clinical trials and meta-analyses to identify probiotic strains with evidence-based clinical indications, and (ii) functional genomic profiling to reconstruct metabolic interaction networks among gut microbial strains [35].

To operationalize this knowledge base, supervised fine-tuning was performed on a pre-trained large language model, DeepSeek, using Low-Rank Adaptation (LoRA). The fine-tuning dataset comprised instruction-output pairs constructed from the knowledge base. Each instruction encoded participant-specific clinical and microbiome features, such as baseline microbial diversity, inferred short-chain fatty acid (SCFA)-related functional deficits, and pathogen/pathobiont burden. The corresponding output generated a candidate synbiotic regimen, including proposed strain selection, rationale for combinations, dosage considerations, and prebiotic/postbiotic pairing.

Importantly, model-generated outputs were used only as auxiliary reference materials to support clinician review. The AI system did not determine, prescribe, or finalize any intervention. All individualized synbiotic formulations and subsequent longitudinal adjustments were independently evaluated, modified when necessary, and confirmed by the responsible physicians.

### 2.5. Personalized Probiotic Intervention Method

#### 2.5.1. Sample Collection and Host Metadata Acquisition

Fecal samples were collected using standardized sterile collection tubes (containing RNAlater RNA Stabilization Reagent, Thermo Fisher, Waltham, MA, USA) and immediately stabilized according to the manufacturer’s protocol. In parallel, multidimensional host metadata were obtained from structured questionnaires and electronic health records, including dietary habits, medication history (with emphasis on recent and/or long-term antibiotic exposure), lifestyle factors, and major gastrointestinal or systemic symptoms, which can be found in Supplementary Files S1. These host-level variables were incorporated as contextual covariates for downstream analysis, including interpretation of metagenomic features and assessment of probiotic responsiveness [36,37].

#### 2.5.2. Shotgun Metagenomic Sequencing

Total microbial DNA was extracted from stool samples using validated fecal DNA extraction kits (QIAamp PowerFecal Pro DNA Kit, Cat# 51804, QIAGEN, Venlo, The Netherlands). DNA purity and concentration were assessed using a NanoDrop One spectrophotometer and a Qubit 4 Fluorometer (Invitrogen, Thermo Fisher, Waltham, MA, USA). DNA integrity was evaluated using an Agilent 4150 TapeStation system (Agilent, Santa Clara, CA, USA) prior to library construction [38]. Sequencing libraries were prepared for shotgun metagenomic sequencing using the TIANSeq Fast DNA Library Prep Kit (TIANGEN Biotech, NG102-01/02, Beijing, China). The libraries were subsequently sequenced on an Illumina NovaSeq 6000 platform (San Diego, CA, USA) in paired-end mode. [39]. Raw sequencing reads were processed through a standardized quality-control workflow, including adapter trimming, removal of low-quality reads, and host-read decontamination via human-sequence filtering. High-quality non-host reads were retained for downstream taxonomic and functional analyses [40].

#### 2.5.3. Taxonomic Structure and Metabolic Potential Profiling

Clean metagenomic reads were analyzed using a two-stage bioinformatics pipeline. Microbial taxonomic composition and relative abundances were quantified using MetaPhlAn4 software, which leverages species-specific marker genes to achieve high-resolution taxonomic classification. This approach enabled characterization of the microbial community structure at the species level, including determination of the overall microbial composition, dominant species, and abundance changes in differential taxa, and supported subsequent analyses of community diversity, commensal microbiota, and structural features of opportunistic pathogens.

Microbial functional annotation and metabolic potential analysis were performed using HUMAnN3 software (Version v3.6). Functional gene annotation was primarily based on the KEGG metabolic pathway database, enabling relative quantification of carbohydrate metabolism, lipid metabolism, amino acid metabolism, short-chain fatty acid (SCFA) biosynthesis capacity (acetate- and butyrate-related modules), inflammation-associated metabolic pathways, and neuroactive metabolite-related pathways [41,42,43]. Additionally, a species–metabolite association mapping strategy was applied to link taxonomic profiles with secondary metabolites and microbe-derived metabolites [44]. The resulting outputs of species abundance and functional gene abundance were transformed, via defined mapping relationships, into indicator values reported in this study and used for subsequent assessments of gut microecological health.

#### 2.5.4. AI-Assisted Comparative Analysis and Dysbiosis Phenotyping

A comparative benchmarking was used to integrate host metadata with metagenomic features and to compare each individual profile against an internal healthy reference baseline comprising over 400 pediatric gut metagenomes from children aged 3–18 years without neurodevelopmental or gastrointestinal disorders. A custom Python (Version 3.9) script was used to compare each participant’s metrics against this healthy reference population, and percentile ranks (0–100th) for each metabolic indicator were calculated [45]. The resulting species and metabolic indicators were summarized into five domains: microbial diversity, abundance of beneficial taxa, pathogen/pathobiont burden, SCFA-related functional potential, and pro-inflammatory features [46]. With the assistance of AI, each participant’s five-domain profile was compared against the healthy reference database to support clinical interpretation. Based on this multidimensional profile, individuals were categorized into a predominant dysbiosis pattern (e.g., low-diversity, SCFA-deficiency, or inflammatory type), which informed the initial intervention strategy [47,48]. Final intervention decisions, including specific strain selection, dosage, and longitudinal adjustments, were made by the clinical team through integration of the AI-assisted comparative outputs, host clinical context, and treatment tolerance [49,50].

### 2.6. Personalized Probiotic Formulation (Function-Matched Strain Combination)

**Figure 2:**
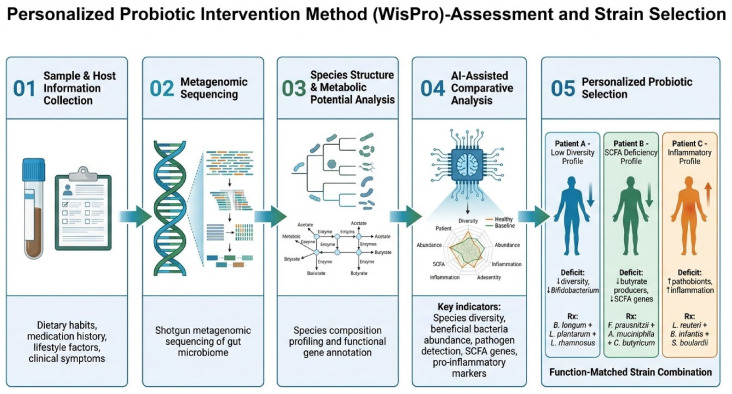
Workflow for personalized probiotic intervention based on metagenomic and host-context analysis.

Probiotic formulations were selected using a function-matched strain-combination strategy rather than generic “beneficial strain” labeling. If the same bacterial species name appears in the formula, it represents different subtypes of the same species. The overall workflow of the personalized probiotic intervention strategy is illustrated in Figure 2.

The intervention pipeline integrates host metadata collection, shotgun metagenomic sequencing, microbial taxonomic and functional profiling, and AI-assisted comparative analysis. Based on these multidimensional data, individual dysbiosis patterns are identified, including low-diversity profiles, short-chain fatty acid (SCFA) deficiency, or inflammatory microbiome signatures. Personalized probiotic formulations are then designed using function-matched strain combinations to restore ecological balance in the gut microbiome.

### 2.7. Design of Personalized Interventions

This personalized intervention comprises three components: the functional screening of probiotic strains, a customized combination of prebiotics and postbiotics, and personalized dietary recommendations. First, the selection of the probiotic strain combination is guided by results from fecal metagenomic analysis. Specific metrics considered include microbial diversity, the relative abundance of beneficial commensal flora, the burden of potential or opportunistic pathogens, and inferred characteristics of metabolic pathways associated with short-chain fatty acid production, bile acid metabolism, and glucose-lipid metabolism. These analytical results guide the probiotic selection process, ensuring the functional attributes of the selected strains align with the predetermined intervention objectives. Second, the selection of prebiotic and/or postbiotic components is based on the subject’s microbiota’s capacity for carbohydrate utilization and the abundance of acid-producing or fiber-fermenting bacteria within their system. Potential prebiotic candidates include fructooligosaccharides (FOS), galactooligosaccharides (GOS), inulin, and resistant starch; the choice depends on the subject’s unique gut microbiome and individual tolerance levels. The use of these components provides targeted nutritional substrates that support the activity of the selected probiotic strains and facilitate the restructuring of the gut microbial community toward a more stable, healthy state. Third, the formulation of personalized dietary recommendations considers the results of the metagenomic analysis, dietary preferences, the subject’s self-reported food sensitivities or intolerances, medical history, and metabolic profile. These dietary recommendations entail avoiding foods that trigger individual intolerance or disrupt microbial balance, while increasing intake of dietary fiber, fermented foods, and specific nutrients compatible with the subject’s unique microbiota. Thereby serving as a non-pharmacological adjunct to microbial modulation therapy. In summary, each intervention plan consists of a personalized probiotic formulation, a customized regimen of prebiotic and/or postbiotic supplements, and a set of personalized dietary guidelines.

The intervention followed a stepped, individualized adjustment strategy. The standard reassessment interval was every 3 months; however, when sample collection was not feasible, reassessment was conducted at approximately 6-month intervals. At baseline, an initial probiotic formulation and prebiotic/postbiotic ratio were selected based on preliminary metagenomic screening results, and individualized dietary recommendations were provided concurrently. At each follow-up assessment, fecal metagenomic profiling was repeated together with evaluation of gastrointestinal symptoms, behavioral outcomes, and available metabolic indicators. If microbial diversity, beneficial taxa, gastrointestinal symptoms, and clinical observations showed favorable or stable changes, the original formulation was maintained or, when appropriate, adjusted to a lower maintenance dose. If microbial improvement was limited, dysbiosis persisted, or inferred functional pathway abnormalities remained, the probiotic strain combination, prebiotic/postbiotic ratio, dosage, and dietary recommendations were adjusted accordingly. This process was repeated across follow-up cycles using the sequence of metagenomic reassessment, microbial phenotype interpretation, formulation adjustment, prebiotic/postbiotic modification, and dose optimization. When microbial and clinical indicators stabilized, participants were transitioned to a lower-dose or fixed-dose maintenance strategy when appropriate.

All participants were instructed to follow a regular daily administration schedule throughout the intervention period. The synbiotic formulation was taken at a fixed time each day on an empty stomach with warm water. Depending on the individualized protocol, administration was scheduled as either a single daily dose or two divided doses in the morning and evening. Participants and caregivers were advised to avoid intermittent use, unscheduled discontinuation, or missed doses whenever possible, except in the presence of specific physiological discomfort, adverse events, or other unforeseen circumstances. This standardized administration schedule was intended to support consistent exposure to the synbiotic formulation and facilitate longitudinal assessment of microbiome and clinical changes. Dietary recommendations, when provided, were also intended to be maintained consistently alongside synbiotic administration during the follow-up period.

### 2.8. Clinical Outcome Assessment

Aberrant Behavior Checklist (ABC) is a validated instrument used to quantify behavioral disturbances in individuals with developmental disorders, including autism spectrum disorder (ASD). Items are rated by severity/frequency; higher scores indicate greater behavioral impairment, with reductions in scores reflecting clinical improvement [51].

The Social Responsiveness Scale (SRS) assesses multiple domains of social functioning, including social awareness, social cognition, social communication, social motivation, and restricted/repetitive behaviors. It is used to quantify the severity of ASD related traits and monitor changes in social functioning, and different versions are available for different age groups. Items are rated on a Likert scale with higher scores indicating greater social impairment [52].

The Clinical Global Impressions Scale (CGI) is a clinician-rated measure used to assess overall illness severity and treatment response over time. It typically consists of two core components: 1. CGI-Severity (CGI-S): rated on a 7-point scale with higher scores indicating greater illness severity; and 2. CGI-Improvement (CGI-I): rated on a 7-point scale, where a score of 4 indicates no change, scores <4 indicate improvement, and scores >4 indicate worsening [53].

## 3. Results

### 3.1. Participant Characteristics

Seven children, who are Case A to Case G with autism spectrum disorder (ASD), were included in this case series. The participants ranged in age from 5 to 12 years and included four males and three females. Six children were classified as having severe ASD, while one child was classified as having moderate ASD.

All participants presented with multiple comorbid conditions, including gastrointestinal dysfunction, allergic disorders, sleep disturbances, and feeding difficulties. Baseline Clinical Global Impression-Severity (CGI-S) scores ranged from 4 to 7. Detailed demographic and clinical characteristics are summarized in Table 1.

For typical subjects undergoing standardized interventions, the standard follow-up period is 6 to 9 months, with the primary endpoints being the preliminary restoration of the gut microbiota and the alleviation of clinical symptoms. Case A, however, presented with a distinct profile characterized by underlying metabolic abnormalities, compromised intestinal barrier function, low baseline microbial diversity, and a susceptibility to recurrent dysbiosis. Although the standard period intervention yielded improvements, the caregivers remained concerned that microbial homeostasis had not yet been fully consolidated and that there was a risk of relapse following the discontinuation of treatment. To assess the long-term stability of the gut microbiota and the sustained efficacy of symptom management under a regimen of prolonged, personalized, and iteratively refined interventions, this study extended the follow-up period for this specific case to 21 months. This extended observation period was used to assess the value of long-term interventions in the management of complex, refractory cases of gut microbiota dysbiosis.

Metabolites and neurochemicals were reported as population percentiles, with the 25th–75th percentile range used as a reference interval. These percentile values represent relative positions within a reference distribution rather than absolute quantitative measurements and may vary depending on developmental stage, diet, and sampling conditions. Therefore, interpretation focused on directional trends and convergence toward the reference interval rather than if higher or lower percentiles necessarily indicate clinical improvement.

### 3.2. Gastrointestinal Outcomes

**Figure 3:**
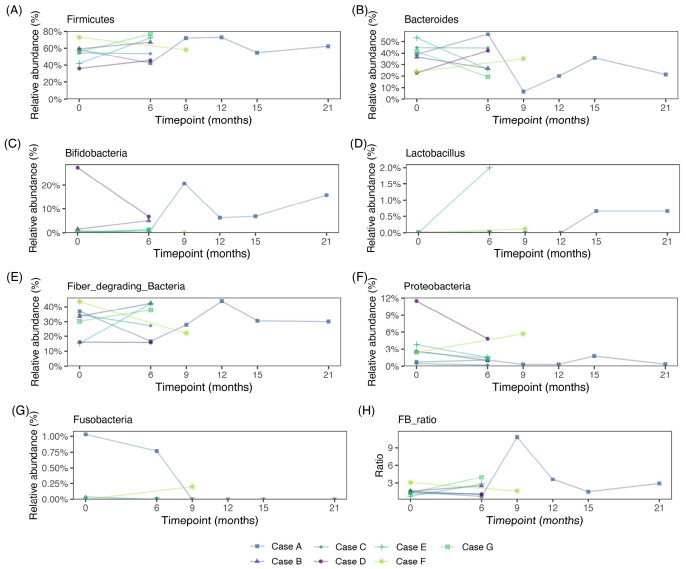
Longitudinal changes in major gut microbial taxa across individual cases. Temporal trajectories of major gut microbial taxa are shown for Cases A-G across follow-up timepoints. Panels depict the relative abundance of (**A**) Firmicutes, (**B**) Bacteroides, (**C**) Bifidobacteria, (**D**) Lactobacillus, (**E**) fiber-degrading bacteria, (**F**) Proteobacteria, and (**G**) Fusobacteria, together with (**H**) the Firmicutes/Bacteroides (F/B) ratio.

Figure 3 shows longitudinal dynamics of major gut microbial taxa across individual cases during personalized microbiome intervention. Each panel shows relative abundance changes in dominant microbial groups over time. Supplementary Table S1 details the changes in the relative abundance of major intestinal microorganisms for each case across different follow-up periods. Supplementary Table S2 shows the baseline synbiotic formulations for everyone, as well as the adjusted formulations implemented following each subsequent assessment of microbial relative abundance. Following microbiome-guided personalized intervention, improvements in gastrointestinal symptoms were observed in several participants. These improvements included normalization of stool frequency and consistency, reduction in stool odor, decreased constipation, and improved digestive tolerance.

The most pronounced gastrointestinal improvements were observed in Cases B, D, and G. Cases A and F demonstrated partial gastrointestinal improvement during the early intervention period, followed by fluctuations over time. Case E showed only minor improvement in constipation symptoms.

In several cases, improvements in sleep quality and reductions in daytime irritability were observed alongside improvements in gastrointestinal function.

Table 2 summarizes these gastrointestinal and behavioral outcomes following personalized microbiome intervention across the seven cases.

### 3.3. Behavioral Outcomes

**Figure 4:**
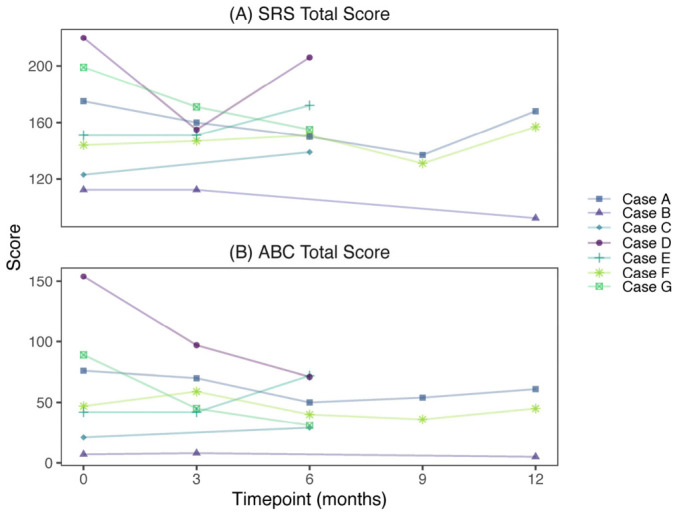
Longitudinal changes in behavioral severity scores following microbiome intervention. Temporal trajectories of behavioral severity scores are shown for Cases A-G across follow-up timepoints. Panels represent changes in (**A**) Social Responsiveness Scale (SRS) total score and (**B**) Aberrant Behavior Checklist (ABC) total score.

Behavioral outcomes were evaluated using the Social Responsiveness Scale (SRS) and the Aberrant Behavior Checklist (ABC). Figure 4 shows trajectories of clinical symptom severity. Supplementary Table S3 shows the behavioral scores of different individuals across various follow-up periods. Behavioral responses demonstrate heterogeneous clinical trajectories among patients.

Cases B, D, and G showed consistent reductions in both SRS and ABC scores during follow-up. Case A demonstrated improvement in behavioral scores up to month 9, followed by partial rebound at month 12. Case F showed a similar trajectory, with early improvement and later fluctuation. Case C showed minimal behavioral change, while Case E demonstrated worsening scores during the observation period.

Behavioral assessment data were not available at all follow-up time points for all participants. In particular, SRS and ABC scores for Cases C, D, E, and G were missing at months 9, 12, and 15 because the participants and caregivers were unable to complete the scales at these follow-up points due to scheduling constraints and the practical burden of repeated questionnaire-based assessments. Missing values were not imputed, and behavioral trajectories were interpreted based only on available observed data.

Overall, behavioral outcomes showed heterogeneous trajectories across participants. 

### 3.4. Neurochemical and Metabolite Changes

**Figure 5:**
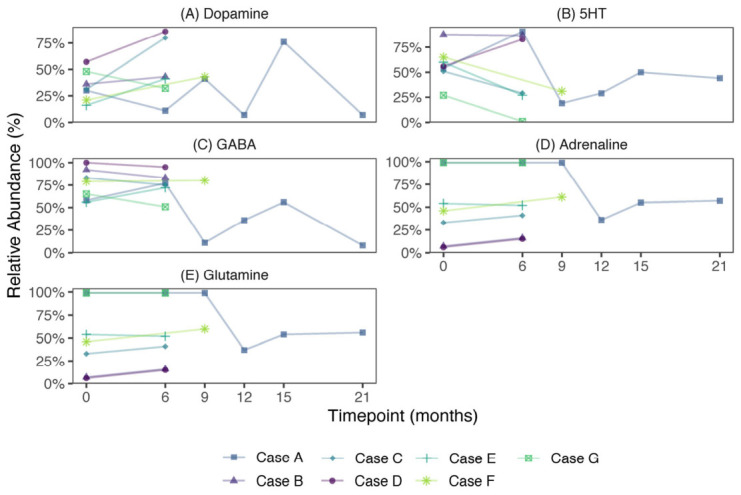
The relative position of temporal percentile changes in neuroactive metabolites during microbiome intervention. Percentile trajectories of major neuroactive metabolites across follow-up timepoints are shown for Cases A-G. Panels represent changes in (**A**) dopamine, (**B**) serotonin (5-HT), (**C**) GABA, (**D**) adrenaline, and (**E**) glutamine.

Neurochemical and metabolite levels were reported as population percentiles. These percentile values represent each participant’s relative position within a reference distribution and should not be interpreted as absolute metabolite concentrations or direct indicators. Changes in these percentiles varied substantially across participants and across time. Figure 5 shows percentile trajectories of key neuroactive metabolites across individual cases during the intervention period, including dopamine, serotonin (5-HT), GABA, adrenaline, and glutamine. Supplementary Tables S4 and S5 present data on changes in metabolites, illustrating the percentile shifts in metabolites across individuals during various follow-up periods. During the follow-up period, the microbiota underwent extensive functional remodeling, and changes in these percentiles varied substantially across participants and across time.

Neurochemical and metabolite percentile trajectories exhibited significant variability across different participants and time points. Among participants who demonstrated behavioral improvement, specifically Cases B, D, and G, behavioral scores decreased; however, their metabolite trajectories were inconsistent. Case A showed behavioral improvement during the early and mid-stages of follow-up but experienced a partial relapse at the final stage, while certain neurochemical percentiles also fluctuated over time. In contrast, Cases C and E, despite longitudinal changes in their metabolite profiles, showed limited behavioral improvement or worsening.

### 3.5. Individual Case Trajectories

Individual clinical trajectories for each participant are summarized below.

Case A was a 5-year-old girl with severe ASD and multiple comorbidities, including eczema, milk allergy, and sleep disturbance. Following personalized microbiome intervention, improvements were observed in gastrointestinal function, eczema symptoms, sleep quality, and daytime mood. Behavioral scores improved until month 9 but showed partial rebound at month 12.

Case B was a 6-year-old boy with a history of perinatal hypoxia and global developmental delay. After intervention, improvements were observed in microbiome diversity, reduction in undigested stool residue, decreased irritability, and improved sleep onset. Both SRS and ABC scores improved over the 12-month follow-up.

Case C was a 6-year-old boy with gluten-sensitive enteropathy and gastrointestinal dysfunction. After intervention, bowel regularity improved, but behavioral scores showed slight increases during the observation period.

Case D was an 8-year-old boy with gastroesophageal reflux disease, enteritis, and a feeding disorder. Following microbiome-guided intervention, harmful bacterial abundance decreased, and sleep quality improved. ABC scores decreased significantly, while SRS scores showed modest improvement.

Case E was a 12-year-old boy with atopic dermatitis and gluten sensitivity. Following the intervention, constipation improved slightly, but stool odor increased. Behavioral scores worsened during the follow-up period.

Case F was a 7-year-old girl with a gluten allergy and persistent loose stools. Early improvements in diarrhea and sleep quality were observed following intervention. However, relapse occurred later with foul-smelling stools and abdominal discomfort. Behavioral scores improved initially but rebounded by month 12.

Case G was a 7-year-old girl with an autoimmune disease and chronic diarrhea. Following the intervention, stool consistency improved, and stool odor decreased. Verbal communication improved, and both SRS and ABC scores decreased significantly.

Concomitant interventions were largely consistent across all participants. Throughout the study period, all children continued to receive their established standard supportive therapies, including Applied Behavior Analysis (ABA), occupational therapy (OT), and speech therapy. These behavioral and developmental interventions remained unchanged before and after the synbiotic intervention. During the observation period, no participants received any other medications. In addition, physician-recommended dietary guidance was provided when clinically appropriate; however, adherence to these recommendations was not uniform across participants during follow-up. Furthermore, participants differed in regional dietary backgrounds, with most from China and one from the United States, leading to variation in staple foods, dairy exposure, gluten-containing foods, fermented food intake, and overall dietary routines. These dietary factors were recorded as contextual variables when summarizing individual case trajectories. No other relevant concomitant interventions were reported.

### 3.6. Response Pattern Summary

**Figure 6:**
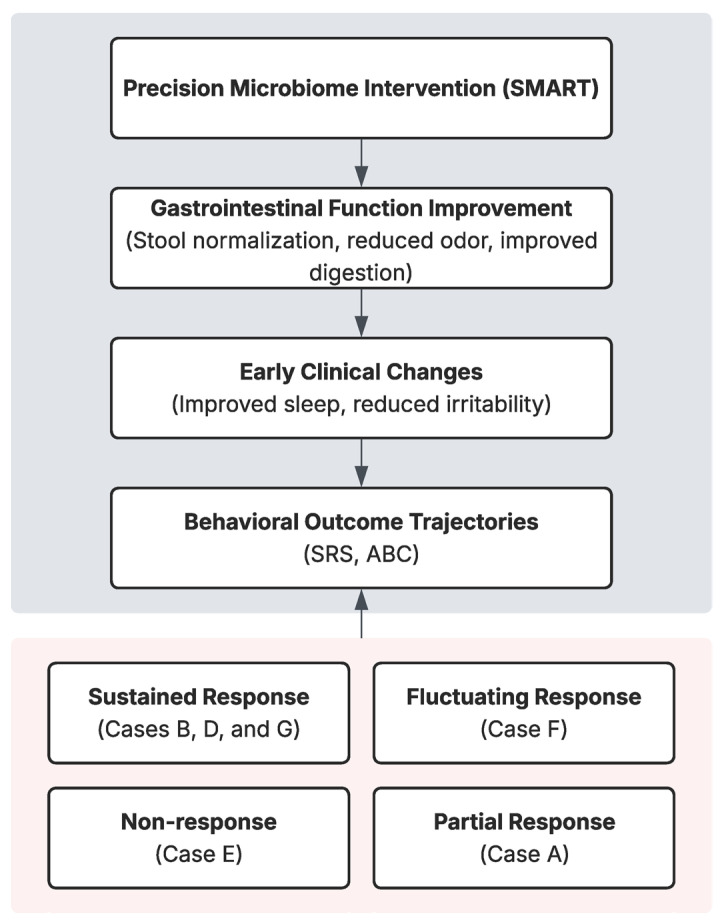
Clinical Response Patterns in ASD Following Precision Microbiome Intervention.

Figure 6 shows three response trajectories: Cases B, D, and G showed a sustained response, with consistent improvements in both gastrointestinal symptoms and behavioral outcomes over time. Cases A and F demonstrated a partial response with fluctuation, characterized by initial improvement followed by later variability in symptom course. In contrast, Case E showed a minimal or negative response, with behavioral outcomes worsening despite the intervention. Together, these patterns highlight the heterogeneity of treatment response across the case series. 

## 4. Discussion

### 4.1. Feasibility and Clinical Utility of an Individualized Approach

This case series demonstrates the feasibility and translational potential of a metagenomics-guided microbiome intervention in children with autism spectrum disorder (ASD). The intervention strategy aimed to selectively restore beneficial microbial taxa while limiting pathogenic overgrowth, thereby tailoring synbiotic formulations to each individual’s microbiome profile and treatment tolerance. Stool samples were collected at multiple time points to enable longitudinal optimization of the intervention strategy. Children with ASD tolerated the synbiotic formulation well; no serious adverse events were observed, and mild adverse reactions (such as transient changes in stool odor or irritability) were self-limited and resolved without intervention.

This individualized strategy seeks to address gastrointestinal and behavioral symptoms by targeting underlying microbial dysbiosis and restoring microbial ecological balance. Previous studies have frequently employed standardized probiotic approaches, in which identical probiotic formulations are administered to all participants, often resulting in variable therapeutic outcomes [28,30,54,55]. In contrast, a precision microbiome strategy leverages metagenomic profiling to guide the rational selection of synbiotic components (probiotics combined with prebiotics), thereby increasing the likelihood that individual patients receive biologically appropriate microbial support tailored to their specific microbial ecosystem.

An additional advantage of this strategy lies in its ability to adapt to the dynamic nature of the gut microbiome. After each follow-up stage, intervention strategies were guided by microbiome sequencing results obtained during the preceding stage. For example, when the microbiota produced increased levels of short-chain fatty acids (SCFAs), the protocol reduced highly fermentable oligosaccharides and adjusted dietary fiber intake accordingly to prevent excessive fermentation. Conversely, when the abundance of a previously depleted microbial taxon normalized, the corresponding probiotic supplementation was reduced, and the strain composition was recalibrated. This adaptive, feedback-driven approach represents a key advantage of precision microbiome-based interventions, as static probiotic regimens cannot readily accommodate dynamic changes within the gut microbial ecosystem [56].

Collectively, these findings provide preliminary support for the feasibility of a metagenomics-guided synbiotic intervention in ASD. Observed outcomes suggest that gut microbial communities may influence neurodevelopment through multiple biological pathways, including microbial metabolite production, immune modulation, and gut–brain signaling mechanisms [57,58]. For instance, microbial metabolites such as short-chain fatty acids (SCFAs) can influence host immune responses, intestinal barrier integrity, and neuronal signaling pathways that may contribute to neurodevelopmental regulation [59,60]. Given the substantial biological heterogeneity observed in ASD, individualized and mechanism-informed therapeutic strategies may represent a promising direction for future interventions [61].

Nevertheless, these findings should be interpreted with caution given the small sample size and exploratory nature of this study. Larger randomized controlled trials will be required to determine efficacy, reproducibility, and long-term safety. Despite these limitations, this study provides a preliminary framework for integrating microbiome-informed decision-making into ASD treatment strategies and supports the broader concept of precision microbiome medicine for neurodevelopmental disorders.

The use of shotgun metagenomic sequencing enabled species-level resolution and functional pathway characterization, enhancing the biological precision of microbiome-guided interventions.

### 4.2. SMART Strategy

Systematic Microbiome Assessment and Reconstruction Therapy (SMART) is a conceptual framework designed to move beyond the traditional “one-size-fits-all” probiotic paradigm. It recognizes that a healthy microbiome is highly context-dependent, shaped by individual diet, environment, and clinical status. In this exploratory case series, we used the SMART framework to organize our intervention approach, rather than to validate the framework itself [62,63,64].

The SMART framework comprises three iterative stages. The first stage involves systematic multidimensional assessment, integrating host metadata (dietary habits, medication history, clinical symptoms) with shotgun metagenomic profiling to characterize each participant’s dysbiosis pattern, including reduced diversity, functional deficits, or pathogen overgrowth [65,66,67,68].

The second stage is sequential ecological reconstruction. Before introducing probiotics, we used personalized dietary and prebiotic guidance to condition the gut environment, aiming to create favorable niches for subsequent strain colonization. Probiotic strains were then selected based on function-matched combinations targeting each individual’s missing metabolic pathways or overrepresented pathobionts, rather than generic “beneficial” labels [69,70].

The third stage involves dynamic monitoring and adaptive management. Periodic metagenomic reassessment and symptom evaluation guided iterative adjustments to probiotic strains, dosages, and prebiotic components. This feedback-driven process allowed the intervention to adapt to each child’s evolving microbial ecology.

Taken together, the SMART framework provided a useful conceptual structure for delivering a personalized, metagenomics-guided synbiotic intervention in this case series. Our preliminary feasibility observations, including the ability to tailor formulations based on longitudinal microbiome data and the heterogeneity of clinical responses, may help inform future refinements of this framework [71]. However, larger controlled studies are needed to evaluate whether such an approach offers reproducible clinical advantages over standardized probiotic regimens.

### 4.3. Gut–Brain Model and Regulatory Volatility

In index case A, the most significant behavioral improvements and metabolic shifts were observed six months after the initiation of the individualized microbiome intervention, including increased levels of acetate, glycine, serotonin, and GABA. These changes suggest that gut microbial metabolites may mediate microbiota–gut–brain communication through neuroimmune and neuroendocrine signaling pathways, potentially influencing serotonergic regulation [24,25].

Levels of adrenaline and glutamine returned to the reference range only at the 12-month mark, suggesting that metabolic regulatory responses associated with stress and amino acids may be more protracted than the early gastrointestinal and behavioral changes observed in Case A. Adrenaline can be interpreted as a peripheral catecholamine related to sympathoadrenal or adrenomedullary stress activity [72]; glutamine is closely linked to intestinal metabolic function, and glutamate-GABA-related neuroactive pathways [73,74]. Furthermore, given the indirect nature of these indicators, these findings should not be interpreted as a normalization of stress-related or neuroactivity-regulating pathways. Delayed changes in these two may indicate that microbiome-directed interventions initially affect intestinal ecology and digestive function, whereas host metabolic and stress-regulatory systems need a longer adaptation period. These shifts also coincided with a rebound in behavioral scores, suggesting that changes in individual metabolites to the reference range do not directly correspond to sustained behavioral improvement.

Similarly, when behavioral scores rebounded at the 12-month mark, dopamine levels decreased, and glycine concentrations increased greatly. These synchronous changes may reflect ongoing metabolic variability during the follow-up period; however, due to the small sample size and other limitations, they do not support conclusions regarding central reward pathways, excitatory-inhibitory regulation, or other neural mechanisms.

The repeated fluctuations in dopamine and GABA levels, together with persistently elevated glycine levels after 12 months, suggest that the fecal metabolite profile may undergo dynamic changes. Microbiota-directed interventions may induce substantial ecological restructuring of microbial communities before a new equilibrium is reached, a well-recognized property of the gut microbial ecosystems. Specifically, the gut microbiome exhibits (i) long-term host-specific stability, with many microbial strains persisting within individuals for extended periods, and (ii) short-term ecological variability, during which the relative abundance of taxa may fluctuate substantially over time [75,76,77].

Collectively, these observations highlight the dynamic changes in indicators related to microorganisms and fecal metabolites during the intervention process. In this context, transient fluctuations during the intervention period should not necessarily be interpreted as failure; rather, they may represent adaptive restructuring processes occurring during microbiome reorganization. Behavioral responses may therefore reflect the trajectory and stability of these microbial and metabolic adjustments. However, these should be regarded as exploratory longitudinal metabolic changes, requiring cautious interpretation; current data do not confirm a direct or causal relationship between the two.

Previous microbiome-based therapeutic studies have similarly reported that improvements in ASD-related behaviors are accompanied by measurable changes in microbial composition and metabolite profiles [78]. But in this exploratory case series, these parallel changes should be regarded as hypothesis-generating rather than as evidence of precise microbiome intervention mechanisms or therapeutic effects.

### 4.4. Neurochemical Percentiles Do Not Map Linearly to Behavior

Across the case series, the correlation between neurochemical percentiles and autism spectrum disorder (ASD) behavioral scales did not follow a direct linear relationship. Behavioral improvements were observed despite persistent extremes in stress-axis indicators. For example, in Case A, improvements in clinical symptoms and scale scores emerged early and peaked around 9 months, whereas adrenaline and glutamine levels remained at the 99th percentile during the 0–9-month period, declining to within the reference range only at 12 months, at which point SRS/ABC scores also temporarily worsened. Similarly, in Case G, SRS/ABC scores improved significantly, while adrenaline/glutamine levels remained at the 99th percentile at both 0 and 6 months. In Case D, despite elevated dopamine and 5-HT levels, GABA levels remained extremely high, and adrenaline/glutamine levels were below the reference range, while ABC scores also improved significantly.

Collectively, these observations may give rise to the following hypothesis: behavioral outcomes are not determined by a single peripheral neurochemical axis, but instead emerge from multi-system interactions, including gastrointestinal function, immune and inflammatory regulation, sleep architecture, and metabolic state. Such multi-axis coupling may enable behavioral improvements even when certain neurochemical measures remain at extreme percentiles.

Furthermore, the interpretation of changes in fecal metabolites should be regarded as exploratory. While an increase in specific taxa may suggest a shift in microbial function toward a healthy gut microbiota profile, these associations do not prove that neurochemical imbalances have been directly corrected. Fecal metabolite markers provide a non-invasive indicator of gut microbial and metabolic activity; however, they may not accurately reflect metabolite concentrations within the central nervous system. Compounds measured in fecal samples may be influenced by diet and host absorption. Consequently, the observed associations among microbial taxa, fecal metabolites, and behavioral changes should be interpreted with caution, as they are hypothesis-generating rather than indicative of causality.

### 4.5. Non-Response to Directional Microbiome Change

Not all children demonstrate improvement following personalized microbiome interventions. Over a six-month period, Case E experienced a deterioration in neurobehavioral outcomes, minimal gastrointestinal benefit, and foul-smelling stools. This may suggest an imbalance in microbial fermentation or metabolic intolerance. Although Case E’s microbiome profile appeared to exhibit potentially beneficial changes, including a reduction in proteobacteria, an increase in fiber-degrading bacteria, and elevated proportions of several metabolism-associated taxa, the participant’s behavioral scores nonetheless deteriorated significantly. One plausible explanation is that while the intervention successfully altered certain microbial characteristics, it failed to address the primary biological drivers underlying the participant’s neurobehavioral symptoms. Case E has a history of atopic dermatitis and gluten sensitivity; then, if inflammatory processes or dietary triggers persist during the follow-up period, microbiome reconstitution may be insufficient to yield behavioral improvements.

An alternative possibility is that the observed microbial shifts resulted in an excessive increase in fermentation activity. An increase in fiber-degrading bacteria is generally considered a potentially beneficial factor, as these microbes may promote the production of short-chain fatty acids (SCFAs) [79]; however, rapid or excessive fermentation may also contribute to gastrointestinal intolerance symptoms, such as changes in stool odor, particularly when fermentation-related metabolites increase [80]. Consequently, the exacerbation of stool odor in Case E may indicate that the intervention altered microbial metabolism in a manner poorly tolerated by the host, even if certain taxonomic shifts appeared beneficial. This distinction indicates that a taxonomic shift toward “beneficial” bacteria does not necessarily equate to an improvement in host-microbiome functional outcomes.

Overall, Case E may suggest a mismatch between the objectives of a microbiome-oriented intervention and the broader biological context of the individual. The lack of success in this case may be attributable to persistent dietary or immunological triggers, limited host tolerance for fermentation-related metabolic changes, or an ASD subtype that responds poorly to gut-targeted modulation. Therefore, personalized synbiotic interventions should not merely aim to increase theoretically beneficial microbial taxa or reduce pathogenic ones; rather, they must also account for the host’s tolerance, dietary exposures, immunological status, and real-time metabolic consequences. Future microbiome-directed interventions may require more conservative dose-escalation regimens and closer monitoring of gastrointestinal tolerability, as well as consideration of discontinuing the intervention in the absence of clinical benefit to prevent potential side effects.

### 4.6. GI-First Response Model

Across the seven cases, the earliest and most consistent improvements were observed in gastrointestinal function, including stool consistency and frequency, stool odor, resolution of constipation or diarrhea, reduced undigested food residue, and decreased abdominal discomfort.

These improvements were frequently accompanied by improvements in sleep onset and maintenance, as well as reduced daytime irritability. This pattern was particularly evident in children with strong treatment responses, including Cases A, B, and D.

Conversely, when gastrointestinal improvements were incomplete or unstable, neurobehavioral improvements were also less pronounced or inconsistent. For example, Case E demonstrated persistent abnormal stool odor and concurrent worsening of SRS/ABC scores.

These observations support a “gastrointestinal-first” response pattern, in which stabilization of digestion represents the earliest and most proximal outcome of microbiome-guided interventions, whereas neurobehavioral improvements emerge later and are typically more gradual.

The gut–brain axis framework provides a potential explanation for this pattern. Gastrointestinal discomfort, constipation, and diarrhea can contribute to sleep disturbance, autonomic nervous system activation, and increased daytime irritability, thereby exacerbating autism-related behaviors reported in caregiver-based rating scales [19].

Following improvement in gastrointestinal symptoms, sleep regulation, and emotional stability may subsequently improve, thereby creating a physiological context that supports subsequent gains in learning, communication, and social engagement.

Accordingly, improvement in gastrointestinal function may serve as an early indicator of intervention response, whereas behavioral scales may reflect longer-term, downstream neurodevelopmental adaptation.

### 4.7. Biological Heterogeneity of ASD and Divergent Clinical Outcomes

Not all cases showed favorable clinical outcomes. For example, Cases C and E showed apparent improvements in gut microbiota composition, including increased beneficial taxa and elevated levels of butyrate-producing bacteria, yet their SRS or ABC scores did not improve and, in some cases, worsened. This discrepancy highlights the biological heterogeneity of ASD and suggests that microbiome improvements alone may not be sufficient to yield neurobehavioral benefits.

Furthermore, this exploratory case series cannot determine the underlying causes of the participants’ gastrointestinal dysfunction and dysbiosis. Baseline intestinal abnormalities may result from the interplay of multiple factors rather than constituting a pathogenic mechanism of ASD itself. Alterations in gut microbiota and gastrointestinal symptoms may instead represent comorbid or exacerbating factors that influence the behavioral manifestations associated with ASD in certain individuals. For instance, gastrointestinal discomfort could contribute to the behavioral problems reported by caregivers. This interpretation may also account for the heterogeneity of responses observed across different cases: children whose behavioral symptoms are closely linked to gastrointestinal instability may be more likely to exhibit behavioral improvements following gut microbiota modulation, whereas those with persistent dietary, immunological, metabolic, or neurodevelopmental factors may experience limited or even negative behavioral responses. These findings underscore the need for future controlled studies to distinguish among the causal roles, contributing factors, and concomitant phenomena of gut microbiota dysbiosis in ASD.

On the other hand, ASD represents a spectrum disorder encompassing multiple biological subtypes, including gut microbiota dysbiosis, immune activation, and metabolic dysregulation [81,82,83]. Such cases may represent subtypes of ASD in which behavioral symptoms are less dependent on gut–brain axis dysfunction and more strongly driven by intrinsic neurodevelopmental processes. Comorbid conditions may influence intervention outcomes. Both children had dietary sensitivities and metabolic vulnerabilities. For example, Case C had gluten-sensitive enteropathy but continued gluten exposure, potentially exacerbating mucosal inflammation and microbiome dysbiosis. Case E had a history of eczema and fungal infections and did not consistently restrict gluten intake.

In such contexts, beneficial microbiome changes may not translate into neurobehavioral improvements due to persistent immune activation or inflammatory signaling.

These findings underscore the importance of ASD subtype stratification in microbiome-based interventions. Such stratification may help predict response and guide more targeted intervention strategies.

### 4.8. Limitations

First, this study is based on a small case series, and the absence of a randomized or placebo-controlled design limits causal inference regarding the observed changes. Furthermore, ASD behavioral symptoms may naturally change over time due to developmental processes, potentially influencing SRS or ABC scores. Also, no microbial therapy has been established as an FDA-approved intervention specifically for autism spectrum disorder or for improving ASD-associated behavioral and gastrointestinal symptoms. Therefore, this study should be considered exploratory rather than established treatments. In this context, the present study is intended to provide preliminary feasibility observations and a conceptual framework for future controlled research, rather than evidence of clinical effectiveness.

Second, the highly individualized nature of the intervention complicates the interpretation of treatment mechanisms. Each child received an individualized combination of probiotic strains, prebiotics, and adjunctive bioactive compounds, making it difficult to determine the specific contribution of individual components.

Third, dietary factors may constitute a significant background variable. Some participants presented with food allergies, food intolerances, or restrictive dietary patterns; however, due to habitual dietary preferences, daily family routines, and the difficulty in modifying food selectivity in children with ASD, they were unable to consistently adhere to dietary avoidance during the follow-up period. Consequently, variations in diet may have influenced the trajectories of gastrointestinal symptoms and microbial changes. Furthermore, regional dietary differences between participants from China and the United States may have contributed to baseline differences in gut microbiota and to differential responses to the interventions. Therefore, these background factors should be considered when interpreting the observed clinical and microbial changes.

Fourth, the preparation of personalized synbiotic formulations required microbiome sequencing and laboratory processing time, resulting in variable follow-up intervals and intervention durations across participants. In addition, SRS and ABC scores were unavailable for several participants at later follow-up time points because caregivers were unable to complete repeated questionnaires within the follow-up schedule. This incomplete longitudinal behavioral dataset limits the ability to fully evaluate behavioral trajectories and compare response patterns across participants. Future studies should use standardized follow-up windows, dedicated assessment visits, and digital or remote questionnaire systems to reduce missing data.

Fifth, assessments based on caregiver-reported questionnaires may be subject to recall bias. Certain domains, such as gastrointestinal symptoms, sleep quality, irritability, dietary adherence, and behavioral changes, rely on caregiver recall, which may be influenced by memory accuracy, expectation effects, and subjective perceptions of improvement. Furthermore, given that caregivers were aware of the intervention and the assessments were not blinded, reported improvements may not fully reflect objective clinical changes. Future studies should employ blinded behavioral assessments and objective digital monitoring tools to mitigate recall bias and enhance the reliability of outcome measurements.

Sixth, the AI-assisted component in this study functioned as a rule-based comparative benchmarking and visualization tool rather than a trained predictive model requiring formal validation. Future studies incorporating validated machine learning algorithms with predefined performance metrics and external validation cohorts are warranted to strengthen the objectivity and scalability of AI-assisted decision support in precision microbiome interventions.

Finally, most children in this study were from China (one from the USA). Cultural and dietary differences across populations can substantially influence gut microbiome composition. Together with the small sample size of seven participants (*n* = 7), this limits the generalizability of the findings across diverse populations.

### 4.9. Future Directions

First, future research should prioritize larger randomized controlled trials incorporating comprehensive microbiome sequencing data and expanded sample sizes. Comparing individualized microbiome-guided interventions with standardized probiotic therapies will help determine causal relationships and identify the factors that influence clinical outcomes. Standardized follow-up schedules and intervention protocols will further enhance the rigor, comparability, and reproducibility of future studies.

Second, future studies should strengthen the objectivity and reliability of clinical outcome measurements. Remote monitoring tools, including wearable activity trackers and digital behavioral monitoring systems, can be integrated to record daily or weekly behavioral changes, thereby generating more continuous and objective behavioral datasets.

Third, future research should aim to identify clinically meaningful ASD subtypes relevant to microbiome-based interventions. Baseline characteristics should be examined to determine whether gastrointestinal phenotypes and microbiome dysbiosis profiles can predict treatment responsiveness. Stratification models based on baseline features and treatment response patterns may enhance the prediction of therapeutic outcomes and enable more targeted intervention strategies.

Finally, the development of standardized and validated microbiome-guided intervention algorithms, including formally evaluated machine learning models, will be important, ensuring that personalized treatment strategies can be applied consistently across clinicians and clinical centers while maintaining the flexibility required for individualized care. Such frameworks may facilitate the translation of precision microbiome strategies into scalable clinical implementation.

## 5. Conclusions

This exploratory case series describes a metagenomics-guided personalized synbiotic intervention in seven children with ASD, using the SMART framework. The findings suggest that this approach is feasible for integrating longitudinal microbiome profiling, individualized synbiotic formulation, and adaptive monitoring within a precision microbiome intervention model. Across several cases, improvements in gastrointestinal function emerged earlier and more consistently than neurobehavioral changes, suggesting a possible “GI-first” pattern of response; however, this observation remains preliminary and requires confirmation in larger controlled studies. Neurochemical percentiles did not map linearly to behavioral outcomes, indicating that fecal metabolic markers should be interpreted cautiously and in the context of broader gastrointestinal, microbial, dietary, and clinical trajectories. Heterogeneity of clinical observations further highlights the biological diversity of ASD and underscores the importance of patient stratification in microbiome-based interventions. Within this context, the SMART framework provides a conceptual model for transitioning microbiome therapeutics from standardized probiotic supplementation toward ecologically informed precision microbiome medicine. Although larger controlled studies are required, these findings suggest that precision microbiome strategies may represent a promising direction for microbiome-based therapies in neurodevelopmental disorders. Future clinical trials should include larger sample sizes, standardized dietary monitoring, blinded outcome assessment, integrated metabolomic and immune measures, and longer follow-up. Then evaluate the clinical utility, reproducibility, and mechanistic relevance of this precision microbiome strategy.

## Figures and Tables

**Table 1:** Summary of Clinical Characteristics and Outcomes in ASD Case Series.

Case	Age (Year)	Sex	ASD Severity	Major Comorbidities	CGI-S (Baseline)	CGI-I (Follow-Up)
A	5	female	Severe	Milk allergy, eczema, sleep disturbance, restrictive food intake disorder.	7	3
B	6	male	Severe	Perinatal hypoxic–ischemic injury, developmental delay, feeding difficulty, GI dysfunction.	6	2
C	6	male	Severe	Gluten-sensitive enteropathy, chronic GI dysfunction, sleep disturbance.	6	5
D	8	male	Severe	GERD, functional constipation, feeding disorder, allergic diathesis.	7	1
E	12	male	Severe	Atopic dermatitis, dermatophytosis, gluten allergy.	6	6
F	7	female	Moderate	Gluten allergy, GI dysfunction, sleep disturbance.	4	5
G	7	female	Severe	Autoimmune disorder, chronic diarrhea, irritability.	7	1

**Table 2:** Summary of Clinical Outcomes Following Personalized Microbiome Intervention.

Case	Main GI Symptoms (Baseline)	GI Outcome	Behavioral Outcome (SRS/ABC)	Response Pattern
A	Constipation, halitosis	GI function improved	Improved until month 9, rebound at month 12	Partial response
B	Undigested food residue	Stool normalized	Significant improvement	Sustained response
C	Constipation, GI dysfunction	Bowel regularity improved	Slight worsening	Limited response
D	Constipation	Improved GI function	ABC improved, SRS slightly improved	Sustained response
E	Constipation	Minimal improvement	Behavioral worsening	Non-response
F	Loose stools	Initial improvement, later relapse	Rebound at month 12	Fluctuating response
G	Chronic diarrhea	Stool normalized	Significant improvement	Sustained response

## Data Availability

The data that support the findings of this study are available from the corresponding author upon reasonable request.

## Supplementary Materials

The following supporting information can be downloaded at: https://www.mdpi.com/article/10.3390/nu18111694/s1. Supplementary Table S1. Longitudinal Changes in Major Gut Microbial Taxa; Supplementary Table S2. Synbiotic Formulas Adapt to Changes in Gut Microbiota; Supplementary Table S3. Longitudinal Changes in Behavioral Scale Scores; Supplementary Table S4. Longitudinal Changes in Metabolite Indicators; Supplementary Table S5. Longitudinal Changes in Microbial Metabolic Indicators. Each supplementary table provides a comprehensive longitudinal dataset, with results categorized by individual case identifiers (Cases A–G) to track specific microbial and clinical trajectories throughout the intervention.

## References

[1] Lord C., Elsabbagh M., Baird G., Veenstra-Vanderweele J. (2018). Autism Spectrum Disorder. Lancet.

[2] Shaw K.A., Williams S., Patrick M.E., Valencia-Prado M., Durkin M.S., Howerton E.M., Ladd-Acosta C.M., Pas E.T., Bakian A.V., Bartholomew P. (2025). Prevalence and Early Identification of Autism Spectrum Disorder Among Children Aged 4 and 8 Years—Autism and Developmental Disabilities Monitoring Network, 16 Sites, United States, 2022. MMWR Surveill. Summ..

[3] Luo Y., Eran A., Palmer N., Avillach P., Levy-Moonshine A., Szolovits P., Kohane I.S. (2020). A Multidimensional Precision Medicine Approach Identifies an Autism Subtype Characterized by Dyslipidemia. Nat. Med..

[4] Feczko E., Balba N.M., Miranda-Dominguez O., Cordova M., Karalunas S.L., Irwin L., Demeter D.V., Hill A.P., Langhorst B.H., Painter J.G. (2018). Subtyping Cognitive Profiles in Autism Spectrum Disorder Using a Functional Random Forest Algorithm. NeuroImage.

[5] Mosconi M.W., Stevens C.J., Unruh K.E., Shafer R., Elison J.T. (2023). Endophenotype Trait Domains for Advancing Gene Discovery in Autism Spectrum Disorder. J. Neurodev. Disord..

[6] Hyman S.L., Levy S.E., Myers S.M., Kuo D.Z., Apkon S., Davidson L.F., Ellerbeck K.A., Foster J.E.A., Noritz G.H., Council on Children with Disabilities, Section on Developmental and Behavioral Pediatrics (2020). Identification, Evaluation, and Management of Children With Autism Spectrum Disorder. Pediatrics.

[7] Wiggins L.D., Rice C.E., Barger B., Soke G.N., Lee L.-C., Moody E., Edmondson-Pretzel R., Levy S.E. (2019). DSM-5 Criteria for Autism Spectrum Disorder Maximizes Diagnostic Sensitivity and Specificity in Preschool Children. Soc. Psychiatry Psychiatr. Epidemiol..

[8] Balasco L., Provenzano G., Bozzi Y. (2020). Sensory Abnormalities in Autism Spectrum Disorders: A Focus on the Tactile Domain, From Genetic Mouse Models to the Clinic. Front. Psychiatry.

[9] Siniscalco D., Schultz S., Brigida A.L., Antonucci N. (2018). Inflammation and Neuro-Immune Dysregulations in Autism Spectrum Disorders. Pharmaceuticals.

[10] Havdahl A., Niarchou M., Starnawska A., Uddin M., van der Merwe C., Warrier V. (2021). Genetic Contributions to Autism Spectrum Disorder. Psychol. Med..

[11] Morton J.T., Jin D.-M., Mills R.H., Shao Y., Rahman G., McDonald D., Zhu Q., Balaban M., Jiang Y., Cantrell K. (2023). Multi-Level Analysis of the Gut–Brain Axis Shows Autism Spectrum Disorder-Associated Molecular and Microbial Profiles. Nat. Neurosci..

[12] Lenroot R.K., Yeung P.K. (2013). Heterogeneity within Autism Spectrum Disorders: What Have We Learned from Neuroimaging Studies?. Front. Hum. Neurosci..

[13] Loth E. (2023). Does the Current State of Biomarker Discovery in Autism Reflect the Limits of Reductionism in Precision Medicine? Suggestions for an Integrative Approach That Considers Dynamic Mechanisms between Brain, Body, and the Social Environment. Front. Psychiatry.

[14] Mesleh A.G., Abdulla S.A., El-Agnaf O. (2021). Paving the Way toward Personalized Medicine: Current Advances and Challenges in Multi-OMICS Approach in Autism Spectrum Disorder for Biomarkers Discovery and Patient Stratification. J. Pers. Med..

[15] Ristori M.V., Mortera S.L., Marzano V., Guerrera S., Vernocchi P., Ianiro G., Gardini S., Torre G., Valeri G., Vicari S. (2020). Proteomics and Metabolomics Approaches towards a Functional Insight onto AUTISM Spectrum Disorders: Phenotype Stratification and Biomarker Discovery. Int. J. Mol. Sci..

[16] Wang J., Ma B., Wang J., Zhang Z., Chen O. (2022). Global Prevalence of Autism Spectrum Disorder and Its Gastrointestinal Symptoms: A Systematic Review and Meta-Analysis. Front. Psychiatry.

[17] Ibrahim S.H., Voigt R.G., Katusic S.K., Weaver A.L., Barbaresi W.J. (2009). Incidence of Gastrointestinal Symptoms in Children With Autism: A Population-Based Study. Pediatrics.

[18] Chaidez V., Hansen R.L., Hertz-Picciotto I. (2014). Gastrointestinal Problems in Children with Autism, Developmental Delays or Typical Development. J. Autism Dev. Disord..

[19] Madra M., Ringel R., Margolis K.G. (2021). Gastrointestinal Issues and Autism Spectrum Disorder. Psychiatr. Clin. N. Am..

[20] Leader G., Abberton C., Cunningham S., Gilmartin K., Grudzien M., Higgins E., Joshi L., Whelan S., Mannion A. (2022). Gastrointestinal Symptoms in Autism Spectrum Disorder: A Systematic Review. Nutrients.

[21] West K.A., Yin X., Rutherford E.M., Wee B., Choi J., Chrisman B.S., Dunlap K.L., Hannibal R.L., Hartono W., Lin M. (2022). Multi-Angle Meta-Analysis of the Gut Microbiome in Autism Spectrum Disorder: A Step toward Understanding Patient Subgroups. Sci. Rep..

[22] Carabotti M., Scirocco A., Maselli M.A., Severi C. (2015). The Gut-Brain Axis: Interactions between Enteric Microbiota, Central and Enteric Nervous Systems. Ann. Gastroenterol..

[23] Petrut S.-M., Bragaru A.M., Munteanu A.E., Moldovan A.-D., Moldovan C.-A., Rusu E. (2025). Gut over Mind: Exploring the Powerful Gut–Brain Axis. Nutrients.

[24] Reigstad C.S., Salmonson C.E., Rainey J.F., Szurszewski J.H., Linden D.R., Sonnenburg J.L., Farrugia G., Kashyap P.C. (2015). Gut Microbes Promote Colonic Serotonin Production through an Effect of Short-chain Fatty Acids on Enterochromaffin Cells. FASEB J..

[25] Silva Y.P., Bernardi A., Frozza R.L. (2020). The Role of Short-Chain Fatty Acids From Gut Microbiota in Gut-Brain Communication. Front. Endocrinol..

[26] Dargenio V.N., Dargenio C., Castellaneta S., Giacomo A.D., Laguardia M., Schettini F., Francavilla R., Cristofori F. (2023). Intestinal Barrier Dysfunction and Microbiota–Gut–Brain Axis: Possible Implications in the Pathogenesis and Treatment of Autism Spectrum Disorder. Nutrients.

[27] Rahim F., Toguzbaeva K., Qasim N.H., Dzhusupov K.O., Zhumagaliuly A., Khozhamkul R. (2023). Probiotics, Prebiotics, and Synbiotics for Patients with Autism Spectrum Disorder: A Meta-Analysis and Umbrella Review. Front. Nutr..

[28] He X., Liu W., Tang F., Chen X., Song G. (2023). Effects of Probiotics on Autism Spectrum Disorder in Children: A Systematic Review and Meta-Analysis of Clinical Trials. Nutrients.

[29] Arnold L.E., Luna R.A., Williams K., Chan J., Parker R.A., Wu Q., Hollway J.A., Jeffs A., Lu F., Coury D.L. (2019). Probiotics for Gastrointestinal Symptoms and Quality of Life in Autism: A Placebo-Controlled Pilot Trial. J. Child Adolesc. Psychopharmacol..

[30] Santocchi E., Guiducci L., Prosperi M., Calderoni S., Gaggini M., Apicella F., Tancredi R., Billeci L., Mastromarino P., Grossi E. (2020). Effects of Probiotic Supplementation on Gastrointestinal, Sensory and Core Symptoms in Autism Spectrum Disorders: A Randomized Controlled Trial. Front. Psychiatry.

[31] Ranjan R., Rani A., Metwally A., McGee H.S., Perkins D.L. (2016). Analysis of the Microbiome: Advantages of Whole Genome Shotgun versus 16S Amplicon Sequencing. Biochem. Biophys. Res. Commun..

[32] Blanco-Míguez A., Beghini F., Cumbo F., McIver L.J., Thompson K.N., Zolfo M., Manghi P., Dubois L., Huang K.D., Thomas A.M. (2023). Extending and Improving Metagenomic Taxonomic Profiling with Uncharacterized Species Using MetaPhlAn 4. Nat. Biotechnol..

[33] Franzosa E.A., McIver L.J., Rahnavard G., Thompson L.R., Schirmer M., Weingart G., Lipson K.S., Knight R., Caporaso J.G., Segata N. (2018). Species-Level Functional Profiling of Metagenomes and Metatranscriptomes. Nat. Methods.

[34] Magnúsdóttir S., Heinken A., Kutt L., Ravcheev D.A., Bauer E., Noronha A., Greenhalgh K., Jäger C., Baginska J., Wilmes P. (2017). Generation of Genome-Scale Metabolic Reconstructions for 773 Members of the Human Gut Microbiota. Nat. Biotechnol..

[35] Machado D., Andrejev S., Tramontano M., Patil K.R. (2018). Fast Automated Reconstruction of Genome-Scale Metabolic Models for Microbial Species and Communities. Nucleic Acids Res..

[36] Norkeweit F., Schlicht K., Rohmann N., Hartmann K., Türk K., Settgast U., Schulte D.M., Gilbert F., Demetrowitsch T., Brix F. (2025). Healthy Lifestyle, Daytime Sleepiness, and Gut Microbiome Composition Are Determinants of Functional Strength in Humans: A Cross-Sectional Study. Sci. Rep..

[37] Ahrens A.P., Hyötyläinen T., Petrone J.R., Igelström K., George C.D., Garrett T.J., Orešič M., Triplett E.W., Ludvigsson J. (2024). Infant Microbes and Metabolites Point to Childhood Neurodevelopmental Disorders. Cell.

[38] Fernández-Pato A., Sinha T., Gacesa R., Andreu-Sánchez S., Gois M.F.B., Gelderloos-Arends J., Jansen D.B.H., Kruk M., Jaeger M., Joosten L.A.B. (2024). Choice of DNA Extraction Method Affects Stool Microbiome Recovery and Subsequent Phenotypic Association Analyses. Sci. Rep..

[39] Gaulke C.A., Schmeltzer E.R., Dasenko M., Tyler B.M., Thurber R.V., Sharpton T.J. (2021). Evaluation of the Effects of Library Preparation Procedure and Sample Characteristics on the Accuracy of Metagenomic Profiles. mSystems.

[40] Duncan A., Koon W., Sidorczuk K., Ponsero A.J., Tiwari S.K., Hildebrand F., Telatin A. (2025). Best Practice in Microbiome Research. Springer Protocols Handbooks.

[41] Bai D., Chen T., Xun J., Ma C., Luo H., Yang H., Cao C., Cao X., Cui J., Deng Y. (2025). EasyMetagenome: A User-friendly and Flexible Pipeline for Shotgun Metagenomic Analysis in Microbiome Research. iMeta.

[42] Quinn-Bohmann N., Wilmanski T., Sarmiento K.R., Levy L., Lampe J.W., Gurry T., Rappaport N., Ostrem E.M., Venturelli O.S., Diener C. (2024). Microbial Community-Scale Metabolic Modelling Predicts Personalized Short-Chain Fatty Acid Production Profiles in the Human Gut. Nat. Microbiol..

[43] Shin A., Xing Y., Waseem M.R., Siwiec R., James-Stevenson T., Rogers N., Bohm M., Wo J., Lockett C., Gupta A. (2025). Microbiota-Short Chain Fatty Acid Relationships Underlie Clinical Heterogeneity and Identify Key Microbial Targets in Irritable Bowel Syndrome (IBS). Sci. Rep..

[44] Sung J., Kim S., Cabatbat J.J.T., Jang S., Jin Y.-S., Jung G.Y., Chia N., Kim P.-J. (2017). Global Metabolic Interaction Network of the Human Gut Microbiota for Context-Specific Community-Scale Analysis. Nat. Commun..

[45] Dawkins J.J., Gerber G.K. (2025). MMETHANE: Interpretable AI for Predicting Host Status from Microbial Composition and Metabolomics Data. Microbiome.

[46] Aslam H., Trakman G., Dissanayake T., Todd E., Harrison P., Alby C., Jabeen T., Gamage E., Travica N., Marshall S. (2026). Dietary Interventions and the Gut Microbiota: A Systematic Literature Review of 80 Controlled Clinical Trials. J. Transl. Med..

[47] Kim H.S., Oh S.J., Kim B.K., Kim J.E., Kim B.-H., Park Y.-K., Yang B.-G., Lee J.-Y., Bae J.-W., Lee C.K. (2024). Dysbiotic Signatures and Diagnostic Potential of Gut Microbial Markers for Inflammatory Bowel Disease in Korean Population. Sci. Rep..

[48] Tannock G.W. (2023). Understanding the Gut Microbiota.

[49] Kumar A., Xu C., Dakal T.C. (2026). Microbiome Based Precision Medicine through Integrated Multiomics and Machine Learning. Microbiol. Res..

[50] Wang J., Cong Y., Tang B., Liu J., Pu K. (2025). Integrative Analysis of Multi-Omics Data and Gut Microbiota Composition Reveals Prognostic Subtypes and Predicts Immunotherapy Response in Colorectal Cancer Using Machine Learning. Sci. Rep..

[51] Aman M.G., Singh N.N., Stewart A.W., Field C.J. (1985). Psychometric Characteristics of the Aberrant Behavior Checklist. Am. J. Ment. Defic..

[52] Chan W., Smith L.E., Hong J., Greenberg J.S., Mailick M.R. (2017). Validating the Social Responsiveness Scale for Adults with Autism. Autism Res..

[53] Busner J., Targum S.D. (2007). The Clinical Global Impressions Scale: Applying a Research Tool in Clinical Practice. Psychiatry.

[54] Zeng P., Zhang C., Fan Z., Yang C., Cai W., Huang Y., Xiang Z., Wu J., Zhang J., Yang J. (2024). Effect of Probiotics on Children with Autism Spectrum Disorders: A Meta-Analysis. Ital. J. Pediatr..

[55] Guidetti C., Salvini E., Viri M., Deidda F., Amoruso A., Visciglia A., Drago L., Calgaro M., Vitulo N., Pane M. (2022). Randomized Double-Blind Crossover Study for Evaluating a Probiotic Mixture on Gastrointestinal and Behavioral Symptoms of Autistic Children. J. Clin. Med..

[56] Walter J., Maldonado-Gómez M.X., Martínez I. (2018). To Engraft or Not to Engraft: An Ecological Framework for Gut Microbiome Modulation with Live Microbes. Curr. Opin. Biotechnol..

[57] Fung T.C., Olson C.A., Hsiao E.Y. (2017). Interactions between the Microbiota, Immune and Nervous Systems in Health and Disease. Nat. Neurosci..

[58] Wang Q., Yang Q., Liu X. (2023). The Microbiota–Gut–Brain Axis and Neurodevelopmental Disorders. Protein Cell.

[59] Dalile B., Oudenhove L.V., Vervliet B., Verbeke K. (2019). The Role of Short-Chain Fatty Acids in Microbiota–Gut–Brain Communication. Nat. Rev. Gastroenterol. Hepatol..

[60] Peng L., Li Z.-R., Green R.S., Holzmanr I.R., Lin J. (2009). Butyrate Enhances the Intestinal Barrier by Facilitating Tight Junction Assembly via Activation of AMP-Activated Protein Kinase in Caco-2 Cell Monolayers. J. Nutr..

[61] Pérez-Cano L., Chenlo S.A., Sabido-Vera R., Sirci F., Durham L., Guney E. (2023). Translating Precision Medicine for Autism Spectrum Disorder: A Pressing Need. Drug Discov. Today.

[62] Zmora N., Zilberman-Schapira G., Suez J., Mor U., Dori-Bachash M., Bashiardes S., Kotler E., Zur M., Regev-Lehavi D., Brik R.B.-Z. (2018). Personalized Gut Mucosal Colonization Resistance to Empiric Probiotics Is Associated with Unique Host and Microbiome Features. Cell.

[63] Davis E.C., Dinsmoor A.M., Wang M., Donovan S.M. (2020). Microbiome Composition in Pediatric Populations from Birth to Adolescence: Impact of Diet and Prebiotic and Probiotic Interventions. Dig. Dis. Sci..

[64] Parizadeh M., Arrieta M.-C. (2023). The Global Human Gut Microbiome: Genes, Lifestyles, and Diet. Trends Mol. Med..

[65] Sizemore N., Oliphant K., Zheng R., Martin C.R., Claud E.C., Chattopadhyay I. (2024). A Digital Twin of the Infant Microbiome to Predict Neurodevelopmental Deficits. Sci. Adv..

[66] Qin Y., Havulinna A.S., Liu Y., Jousilahti P., Ritchie S.C., Tokolyi A., Sanders J.G., Valsta L., Brożyńska M., Zhu Q. (2022). Combined Effects of Host Genetics and Diet on Human Gut Microbiota and Incident Disease in a Single Population Cohort. Nat. Genet..

[67] Berryman M.A., Milletich P.L., Petrone J.R., Roesch L.F.W., Ilonen J., Triplett E.W., Ludvigsson J. (2022). Autoimmune-Associated Genetics Impact Probiotic Colonization of the Infant Gut. J. Autoimmun..

[68] Xu X., Ocansey D.K.W., Hang S., Wang B., Amoah S., Yi C., Zhang X., Liu L., Mao F. (2022). The Gut Metagenomics and Metabolomics Signature in Patients with Inflammatory Bowel Disease. Gut Pathog..

[69] Gagliardi A., Totino V., Cacciotti F., Iebba V., Neroni B., Bonfiglio G., Trancassini M., Passariello C., Pantanella F., Schippa S. (2018). Rebuilding the Gut Microbiota Ecosystem. Int. J. Environ. Res. Public Health.

[70] Hmar E.B.L., Paul S., Sharma H.K. (2024). An Insight into the Combination of Probiotics and Their Implications for Human Health. Endocr. Metab. Immune Disord. Drug Targets.

[71] Xiong R. (2025). Advancing Digital Precision Medicine for Chronic Fatigue Syndrome through Longitudinal Large-Scale Multi-Modal Biological Omics Modeling with Machine Learning and Artificial Intelligence. arXiv.

[72] Carter J.R., Goldstein D.S. (2021). Sympathoneural and Adrenomedullary Responses to Mental Stress. Compr. Physiol..

[73] Kim M.-H., Kim H. (2017). The Roles of Glutamine in the Intestine and Its Implication in Intestinal Diseases. Int. J. Mol. Sci..

[74] Andersen J.V. (2025). The Glutamate/GABA-Glutamine Cycle: Insights, Updates, and Advances. J. Neurochem..

[75] Lozupone C.A., Stombaugh J.I., Gordon J.I., Jansson J.K., Knight R. (2012). Diversity, Stability and Resilience of the Human Gut Microbiota. Nature.

[76] Flores G.E., Caporaso J.G., Henley J.B., Rideout J.R., Domogala D., Chase J., Leff J.W., Vázquez-Baeza Y., Gonzalez A., Knight R. (2014). Temporal Variability Is a Personalized Feature of the Human Microbiome. Genome Biol..

[77] Faith J.J., Guruge J.L., Charbonneau M., Subramanian S., Seedorf H., Goodman A.L., Clemente J.C., Knight R., Heath A.C., Leibel R.L. (2013). The Long-Term Stability of the Human Gut Microbiota. Science.

[78] Kang D.-W., Adams J.B., Coleman D.M., Pollard E.L., Maldonado J., McDonough-Means S., Caporaso J.G., Krajmalnik-Brown R. (2019). Long-Term Benefit of Microbiota Transfer Therapy on Autism Symptoms and Gut Microbiota. Sci. Rep..

[79] Barber T.M., Kabisch S., Pfeiffer A.F.H., Weickert M.O. (2020). The Health Benefits of Dietary Fibre. Nutrients.

[80] Fernández-Bañares F. (2022). Carbohydrate Maldigestion and Intolerance. Nutrients.

[81] Xu X.-J., Lang J.-D., Yang J., Long B., Liu X.-D., Zeng X.-F., Tian G., You X. (2023). Differences of Gut Microbiota and Behavioral Symptoms between Two Subgroups of Autistic Children Based on γδT Cells-Derived IFN-γ Levels: A Preliminary Study. Front. Immunol..

[82] Matta S.M., Hill-Yardin E.L., Crack P.J. (2019). The Influence of Neuroinflammation in Autism Spectrum Disorder. Brain Behav. Immun..

[83] Frye R.E., Rincon N., McCarty P.J., Brister D., Scheck A.C., Rossignol D.A. (2024). Biomarkers of Mitochondrial Dysfunction in Autism Spectrum Disorder: A Systematic Review and Meta-Analysis. Neurobiol. Dis..

